
JOURNAL INFORMATION
==============================
NLM Title Abbreviation: Lancet
Journal ID: Lancet
NLM Journal ID: 2985213R

Lancet (London, England)

ISSN: 0140-6736
EISSN: 1474-547X

ARTICLE INFORMATION
==============================
PMCID: PMC11222916
PMID: 38521559
DOI: 10.1016/S0140-6736(24)00493-8
Article ID: S0140-6736(24)00493-8
Article version: 1
Subjects: Department of Error

Department of Error

Print publication date: 2024 Mar 23
Volume: 403
Issue: 10432
First page: 1140
Last page: 1140
Copyright: © 2024 The Author(s). Published by Elsevier Ltd.
Copyright year: 2024
Copyright holder: Elsevier Ltd
License: This is an open access article under the CC BY license (http://creativecommons.org/licenses/by/4.0/).
License URL: https://creativecommons.org/licenses/by/4.0/

Erratum for: Severe COVID-19 outcomes after full vaccination of primary schedule and initial boosters: pooled analysis of national prospective cohort studies of 30 million individuals in England, Northern Ireland, Scotland, and Wales 400 10360 14 10 2022 1305 1320 Lancet (London, England) 10.1016/S0140-6736(22)01656-7 PMC9560746 36244382

==============================
Agrawal U, Bedston S, McCowan C, et al. Severe COVID-19 outcomes after full vaccination of primary schedule and initial boosters: pooled analysis of national prospective cohort studies of 30 million individuals in England, Northern Ireland, Scotland, and Wales. Lancet 2022; 400: 1305–20—In this Article, owing to a weighting error in the original analysis, all data related to Northern Ireland have been updated in the abstract, the main text, and tables 1 and 2 (which have been replaced). The numbers of events, rate data, and key findings, conclusions, and interpretation are unchanged. These corrections have been made to the online version as of March 21, 2024.

